# Supplementary material for: Splice-Junction-Based Mapping of Alternative Isoforms in the Human Proteome
Source: Cell Rep. Author manuscript; Available in PMC 2020 Jan 15. (PMC6961840; doi:10.1016/j.celrep.2019.11.026)
Supplement: 2 [file NIHMS1546469-supplement-2.zip › DF1/PXD010154/Pancreas/Pancreas_10_SLC25A39_LLGGCSADSTLSPLQQMVASGTGAVVTSLFMT.pdf]

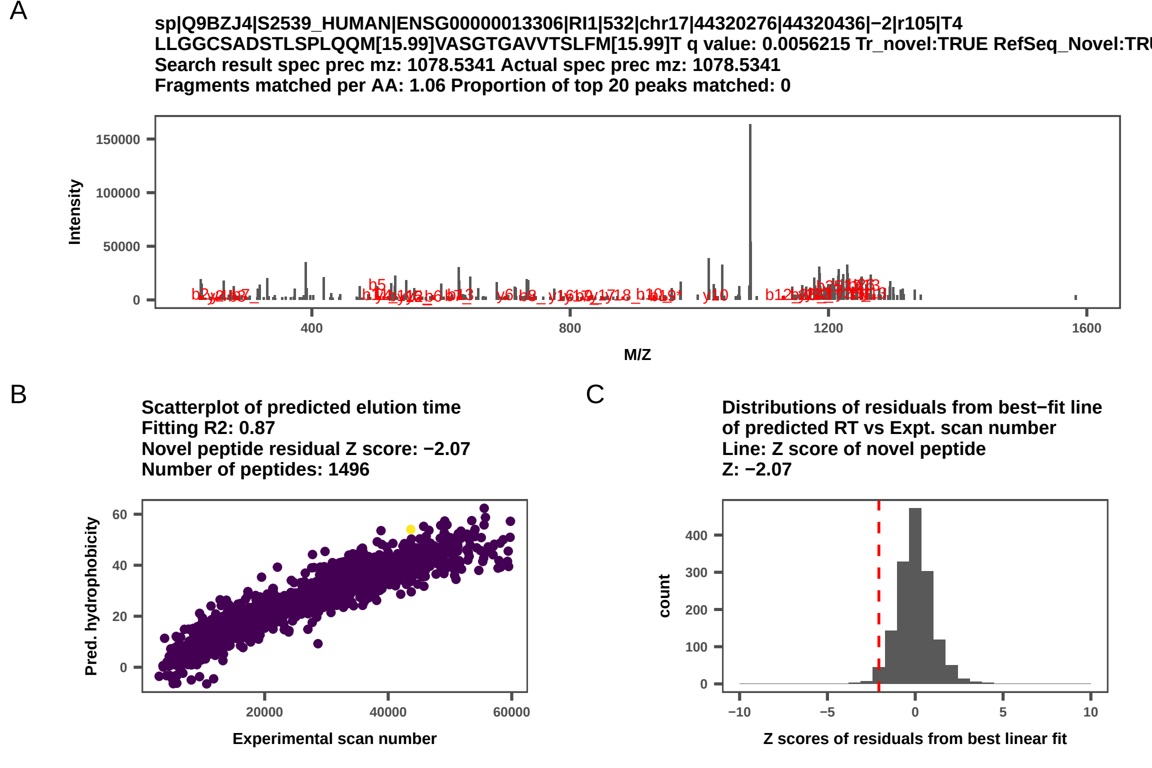
A
sp|Q9BZJ4|S2539_HUMAN|ENSG00000013306|RI1|532|chr17|44320276|44320436|−2|r105|T4
LLGGCSADSTLSPLQQM[15.99]VASGTGAVVTSLFM[15.99]T q value: 0.0056215 Tr_novel:TRUE RefSeq_Novel:TRU
Search result spec prec mz: 1078.5341 Actual spec prec mz: 1078.5341
Fragments matched per AA: 1.06 Proportion of top 20 peaks matched: 0
Intensity
150000
100000
50000
0
400
800
1200
1600
M/Z
B
Scatterplot of predicted elution time
Fitting R2: 0.87
Novel peptide residual Z score: −2.07
Number of peptides: 1496
Pred. hydrophobicity
60
40
20
0
20000
40000
60000
Experimental scan number
C
Distributions of residuals from best−fit line
of predicted RT vs Expt. scan number
Line: Z score of novel peptide
Z: −2.07
count
400
300
200
100
0
−10
−5
0
5
10
Z scores of residuals from best linear fit
